# Hemoglobin Status and Externalizing Behavioral Problems in Children

**DOI:** 10.3390/ijerph13080758

**Published:** 2016-07-26

**Authors:** Jianhua Su, Naixue Cui, Guoping Zhou, Yuexian Ai, Guiju Sun, Sophie R. Zhao, Jianghong Liu

**Affiliations:** 1 Department of Neurology, The Jintan Hospital Affiliated to Jiangsu University, 16 Nanmen Street, Jintan, Changzhou 213200, China; sujianhua961211@sina.com (J.S.); zhou.guoping@hotmail.com (G.Z.); ayx5096@163.com (Y.A.); 2 Schools of Nursing and Medicine, University of Pennsylvania, 418 Curie Blvd., Room 426, Claire M. Fagin Hall, Philadelphia, PA 19104, USA; naixuec@nursing.upenn.edu (N.C.); sophie.r.zhao@hotmail.com (S.R.Z.); 3 Department of Nutrition and Food Hygiene, School of Public Health, Southeast University, Room #508, 87, DingJiaqiao, Nanjing 210096, China; guiju.sun@gmail.com

**Keywords:** aggression, attention problems, Chinese children, externalizing behavior, hemoglobin

## Abstract

*Background*: Still considered one of the most prevalent nutritional problems in the world, anemia has been shown in many studies to have deleterious effects on neurobehavioral development. While most research efforts have focused on investigating the effects of anemia on social and emotional development of infants by using a cross-sectional design, research is still needed to investigate whether early childhood anemia, beyond infantile years, is linked with behavioral problems. *Objective*: This study assessed whether (1) hemoglobin (Hb) levels in early childhood are associated with externalizing behavior; and (2) this relationship is confounded by social adversity. *Methods*: Hemoglobin levels were taken from children (*N* = 98) of the China Jintan Cohort Study at age 4 years, and externalizing behaviors (attention and aggression) were assessed with the Child Behavior Checklist (ASEBA-CBCL) at age 6 years (mean age 5.77 ± 0.39 years old). *Results*: Compared with other children in the sample, children with relatively lower Hb levels at age 4 had more behavioral problems in both attention and aggression at age 6, independent of social adversity. For boys, this association was significant for attention problems, which did not interact with social adversity. For girls, the association was significant for aggression, which interacted with social adversity. While girls on average exhibited higher social adversity than boys, the main effect of Hb was only significant in girls with low social adversity. *Conclusions*: These results indicate that there is an inverse association between hemoglobin levels and later behavioral problems. Findings of this study suggest that regular monitoring of children’s hemoglobin levels and appropriate intervention may help with early identification of behavioral problems.

## 1. Introduction

Despite decades of successful prevention and intervention efforts, anemia is still considered one of the most prevalent nutritional problems in the world. According to one report [1], children under 5 years of age are largely affected by anemia, particularly in countries in Latin America, the Caribbean, the Middle East, and East Asia. Over half of preschool-aged children in southern Asian and African countries are found to be anemic [1,2,3]. A 2004 survey of Chinese children showed that the prevalence of anemia ranged from 8% to 21% in infants up to 36 months old [4]. Young children and pregnant women are particularly at risk for the adverse effects of anemia because childhood and fetal development, respectively, are periods of crucial development of the central nervous system in which biochemical processes—such as myelin genesis and synapse formations—occur. As shown in a number of animal and human studies, infancy and early childhood are periods during which major development of the hippocampus takes place [5,6,7]. Disruption of neurological development processes can lead to decreased cognitive development, in addition to reduced motor activity and thermoregulation [8]. Anemia can be caused by a variety of factors, such as reduced hemoglobin (Hb) levels. Another contributing factor is iron deficiency. Iron, a major functional component of Hb, plays an important role in neurological function by serving as a co-enzyme in the production and release of neurotransmitters [8,9,10,11] and influencing cognitive function [11,12,13,14] and behavioral disorders such as ADHD [15,16].

The deleterious effects of early anemia on neurobehavioral development have been shown to occur throughout the lifespan. In addition to cognitive impairments, altered behaviors have been shown to be among the most troublesome outcomes of anemia in young children [17]. A number of studies have reported behavioral differences in anemic infants, including fatigue, fearfulness, and proximity to the mother [11,18,19,20]. Many manifestations of behavioral abnormalities can be observed in the forms of irritability, restlessness, inattention, and hyperactivity [12,20,21,22]. Similar adverse developmental effects can also be observed among preschool-aged children. In a French study of 4-year-old children, Hb levels were found to be positively associated with developmental, motor, and social quotients, after controlling for socioeconomic factors [23]. Lozoff et al. [24] also reported that anemic Indian preschool children were slower to show positive affect, were more fearful towards new toys, and displayed less social looking towards their mothers than their non-anemic counterparts. Additionally, research has also provided evidence for the long-term effects of infant anemia in school-aged children and adolescents. In a recent longitudinal study by Galler et al. [25], it was found that previously malnourished children, who exhibited low Hb levels, among other signs of malnutrition, showed higher parent-reported levels of aggression toward peers at ages 9–15 years than at 11–17 years, independent of baseline age, sex, household standard of living, and maternal depressive symptoms. In a 10-year longitudinal study in Costa Rica, a school-aged follow-up using the Child Behavior Checklist (CBCL) [26] showed that both parents and teachers of formerly anemic children reported them to have more social and attention problems, particularly internalizing problems like anxiety, depression, withdrawal, and somatic complaints [17]. Malnutrition, including low Hb levels, has been linked to children’s behavioral problems in many other studies around the world. A very recent study by Galler et al. [27] even tracks effects of early malnutrition (including low Hb levels) into adulthood with manifestations of ADHD symptoms. Externalizing behavioral problems, in particular, are risk factors for later juvenile delinquency and violence [28,29,30,31,32]. However, it is worth noting here that most existing research focuses on anemia that was assessed during the infant or toddler years.

Research on the link between anemia and behavioral problems in children has several potential limitations. As most research efforts have been dedicated to investigating the effects of anemia on social and emotional development of infants, it is not clear whether early childhood anemia, beyond infantile years, is linked to behavioral problems. Additionally, despite the fact that the developing area of East Asia is among the regions most affected by childhood anemia, very little evidence exists, to our knowledge, that links anemia to adverse behavioral outcomes of children in this region. Anemia can be caused by decreased iron and/or decreased hemoglobin. However, a previous study found that low iron levels alone did not have a significant association with adverse behavioral outcomes [33]. Consequently, the present study focuses on child hemoglobin levels and childhood behavior. In this study, we use a subset of a large-cohort longitudinal data from Jintan, China in order to address the above-listed gaps in the literature. Our study takes into account 11 different measures of social adversity and seeks to answer the questions of (1) whether Hb levels in early childhood are associated with externalizing behaviors in preschool Chinese children and (2) whether this relationship is confounded by social adversity.

## 2. Methods

### 2.1. Study Site and Participants

As part of the China Jintan Child Health Project cohort, 106 children, 58.2% boys and 41.8% girls, in preschool participated in this study. In China, preschools (where they are called kindergarten) are administrated by the City Maternal-Child Health Center, which is a division of the City Health Department. As part of the public health work for the children in the city, the Jintan Maternal-Child Health Center periodically goes to the preschool to provide health education and health check-ups for children. Jintan Maternal Child Health Center provided annual physical examinations for local preschool children. Assessing hemoglobin levels was part of the routine physical examination. This study utilized the complete sample for both hemoglobin levels and behavioral measures from one of the urban preschools [34,35]. All of the children in the sample of this paper were no older than 6 years at the time of behavior analysis, as children who are older than 6 are not deemed suitable for preschool CBCL [26,36]. Out of the 106 children, 98 have complete data on the three key variables (Hb levels, behavior, and social adversity) and were included in the analyses. Further detailed information on the subjects, recruitment, and setting are given elsewhere [35,37]. Institutional Review Board approval was obtained from the University of Pennsylvania (IRB#8, 811114) and the ethical committee for research at Jintan Hospital in China.

### 2.2. Blood Hb Levels at Age 4 Years

Specimens for blood analysis were collected during the fall 2004 when the children were on average 4 years old. The collection was carried out by trained pediatric nurses using a strict research protocol, in which approximately 0.5 mL of venous blood was collected in a lead-free EDTA tube to avoid contamination. Blood Hb concentration was measured by the 7–22 photoelectric colorimeter at the Jintan Maternal Child Health Center.

### 2.3. Behavioral Assessment at Age 6 Years

Childhood behavioral problems were measured with the Chinese version of the Achenbach System of Empirically Based Assessment (ASEBA) CBCL/1.5–5 [26,36]. Behavior assessment took place in spring 2007 when the children were around 6 years old (mean age 5.77 ± 0.39 years old). The CBCL is a widely used scale for assessing behavioral and emotional problems in children. In this study, parents were asked to answer the 99 items of the CBCL, which dealt with their children’s behavior within the past 12 months, and give a rating from a 3-point scale (0 = not true, 1 = sometimes true, or 2 = often true) [26,36]. One of two broadband factors is externalizing behaviors. Separately, factor analysis has produced two syndromes for externalizing behaviors: attention problems and aggressive behavior [26,36]. These factor structures have also been validated in our previous study [38]. The internal reliabilities (coefficient alpha) for the scales in our study sample were as follows: attention problems (0.64) and aggression (0.87). In this study, we utilized the full externalizing behavior scale with all 24 items. The two subscales of externalizing behavior, attention problems and aggression, were used in subsequent analyses. Complete data on both the blood Hb and behavior variables were available on the 100 subjects.

### 2.4. Social Adversity

Parents were asked to fill in a socio-demographic questionnaire at the same time that they completed the CBCL, when children were 6 years old. The social adversity index was created along lines similar to those previously described by Rutter et al. [39] and Moffitt [40]. A total social adversity score was derived based on 11 variables. This score was created by adding one point (for 9 of the 11 indicators) or two points (for the other 2 indicators) for each of the following 11 social adversity variables: mother’s low education (below middle school—1.0%), father’s low education (below middle school—2.0% ), mother’s low occupational status (3-point scale: 0, professional or skilled work—37.1%; 1, unskilled worker—45.4%; 2, no occupation—17.5%), father’s low occupational status (3-point scale: 0, professional or skilled work—47.4%; 1, unskilled worker—52.6%; and 2—no job, 0.0%), mother’s poor health status (1.0%), father’s poor health status (6.1%), obstetric complication (bleeding, hypertension, diabetes, Caesarian-section, difficult birth, low birth weight, difficulty breathing—56.7%); divorce (4.2%), absence of biological mother (7.0%), house size below 70 square meters (7.1%), and poor neighborhood (overcrowded neighborhood, noise pollution, damp—74.0%). The social adversity score ranged from 0 to 11 (M = 3.07, SD = 2.06). The social adversity score therefore ranges from 0 to 11, where higher scores indicates more counts of social adversity. We used a 50th percentile cut point (3 points or more) to establish a dichotomy of high–low social adversity. Complete data for this variable and blood Hb was available on 98 children. It should be noted that the social adversity in the present study did not include negative life events like child abuse, witnessing violence, or any other childhood trauma.

### 2.5. Statistical Analysis

At age 4 years, 98 children had complete data on all independent and dependent variables. Table 1 details the means and standard deviations of all key variables used in the analyses.

To test for the overall effects of Hb levels on the two measures of externalizing behavior (attention problems and aggression), univariate and multivariate analysis of variance (ANOVA and MANOVA, respectively) were conducted using IBM SPSS 19 (IBM Corporation 2010, Armonk, NY, USA).

Hemoglobin levels are classified into three groups with the cutoffs at the 25th and 75th percentiles. Due to our small sample size (98 children overall with complete data), the three-group classifications for Hb levels were used instead of two-group (low-Hb/high-Hb with cut off at 12.5 g/dL according to the clinical cutoff for anemia [41]) in ANOVAs and MANOVAs in order to better understand the link between Hb and externalizing behavior. MANOVA aims to assess the main effect of Hb levels on total externalizing behavior, while univariate ANOVA further clarifies the association by identifying which of the externalizing behavior subscales is particularly related to Hb.

As we expect behavior to be influenced by social adversity, we tested for moderating effects of social adversity by recoding the 11-point adversity score into categorical variables of high vs. low social adversity, with cutoff set at the 50th percentile (3.0 social adversity score). The categorical social adversity variable was then entered into the ANOVAs and MANOVAs along with Hb groups and interaction effects. To assess the moderating effect of sex, we entered the variable as a factor in the ANOVAs and MANOVAs along with Hb groups and interaction effects. Two-tailed tests of significance are used throughout the analyses and significance is defined as two-tailed *p* value ≤ 0.05 and trending significance is defined as two tailed *p* value higher than 0.05 but less or equal to 0.01.

## 3. Results

Table 2 shows the demographic measures among children stratified by hemoglobin levels at age 4. Detailed results of the ANOVAs and MANOVAs of the effects of Hb levels at age 4 on externalizing behaviors at age 6 are shown in Table 3, along with results from moderator analyses.

### 3.1. Effect of Low Hb

**Figure 1:**
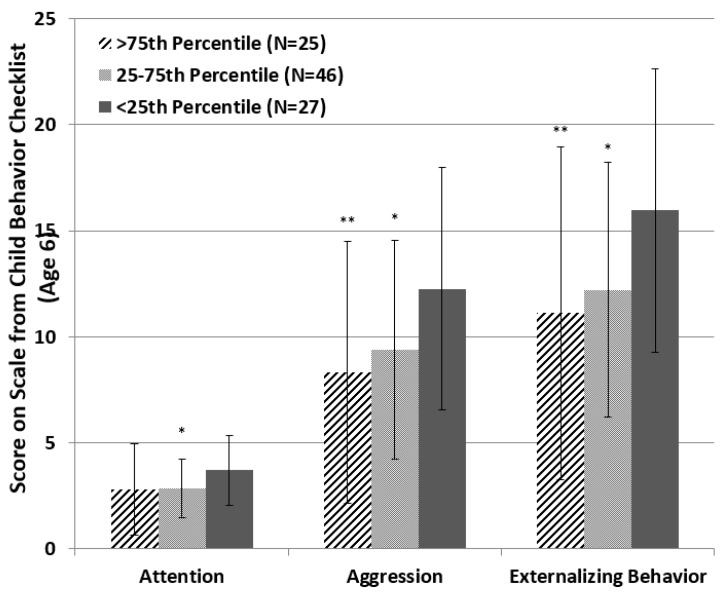
Score for externalizing behaviors at age 6 among children who tested for hemoglobin levels at age 4. * Trending significant (0.05 < *p* ≤ 0.1) differences in behavior score when compared with the <25th percentile group. ** Significant (at α = 0.05) differences in behavior score when compared with the <25th percentile group.

Our results show that at age 4, children in the low Hb group—the lower 25th percent of the data—consistently scored higher on all externalizing behavior scales (Figure 1). There were significant differences in aggression and total externalizing behavior scores between the higher than 75th percentile and lower than 25th percentile Hb groups; there was trending toward significant differences in attention, aggression, and total externalizing behavior scores between the 25th–75th percentile and lower than 25th percentile Hb groups. MANOVA results indicated a significant or trending toward significant main effect from Hb levels at age 4 (Table 3). ANOVA results on attention problems and aggression also indicated individually significant or trending toward significant main effect on Hb levels at age 4, indicating that children in the lower 25th percentile Hb group at age 4 had more problems in both attention and aggression at age 6.

### 3.2. Moderator Effect: Social Adversity-by-Hb Level Interactions

No moderator effect in social adversity was observed in MANOVA and ANOVAs (Table 3). More importantly, after including social adversity and its interaction with Hb as explanatory variables in the analysis, the main effect of Hb on behavioral problems in all the MANOVA (F4, 184 = 2.002, *p* = 0.096) and ANOVAs (attention: F2, 92 = 3.692, *p* = 0.029; aggression: F2, 92 = 2.250, *p* = 0.011) remained significant or trending toward significant. This indicates that the relationships between lower Hb and all externalizing problems are independent of social adversity.

### 3.3. Moderator Effect: Sex-by-Hb Level Interactions

We observed significant interaction effects between sex and Hb levels for attention problems (Table 3), and in order to clarify this interaction, the sample was stratified by sex, and ANOVA and MANOVA were conducted separately for boys and girls (Table 4).

MANOVA showed that the main effect of Hb on externalizing behavior is trending significant for girls, which shows interaction effects in social adversity (Table 4).

ANOVA results indicate that the main effect of Hb in boys is significant in attention problems, which is not moderated by social adversity (Table 4). In girls, however, ANOVA results indicate that the main effect of Hb is only significant in aggression; this main effect is moderated by social adversity (Table 4).

### 3.4. Social Adversity-by-Hb Level Interactions in Girls

In the sex-stratified analyses, we observed significant and trending toward significant social adversity interactions in girls in all of the ANOVA and MANOVAs. To investigate this interaction, we further stratified girls by social adversity and ANOVA and MANOVA were conducted separately for girls with high and low social adversity scores (Table 5). MANOVA showed that the main effect of Hb is only significant in girls with low social adversity scores. ANOVA results indicate that the main effects of Hb in girls with low social adversity are also significant for both subscales of externalizing behavior. To further clarify the direction of the associations found in MANOVA and ANOVAs, post-hoc Tukey tests were conducted on Hb groups, and the results confirmed that within girls with low social adversity, those who had higher Hb levels had lower scores on both aggression and attention problems.

## 4. Discussion

The first key finding in this longitudinal study of Chinese preschool children is that Hb levels at age 4 are negatively associated with externalizing behavioral problems at age 6. This result is concurrent with previously discovered results that anemic infants and children demonstrate behavioral abnormalities [17,20,22,24,42,43,44,45], although mostly manifested through affective changes such as unhappiness and wariness of surroundings. Secondly, we can conclude from our analyses that the observed Hb-externalizing behavior link is not confounded by social adversity. While there have been many papers in the past which investigated the link between more broad spectrum effects of early malnutrition and externalizing behaviors in children [25,46,47], our study adds to the literature on the link between Hb and externalizing behaviors.

Although the mechanism by which low Hb levels influence behavioral and cognitive development is not clearly defined, previous studies have found that children who were anemic infants exhibited anatomical defects such as decreased myelination in the auditory and visual systems of the brain [48,49] as well as neuro-endocrine abnormalities such as alterations in neurotransmitter functions. These abnormalities are particularly prominent in the dopamine system [9,10,11,50], which is crucial in regulating mood, attention, and voluntary movement, and the hippocampus [8,42], an integral component of the limbic system which regulates short- and long-term memory and spatial navigation. These perturbations in the central and autonomic nervous system can then lead to decreased cognitive abilities and behavioral abnormalities.

The third key finding of our study is that there are sex differences in the link between Hb and externalizing behavior. To our knowledge, very few studies on the subject have highlighted sex differences, although it is not clear whether this is because sex differences truly did not exist in previous studies or if there was simply not enough data to support the conclusion. For the boys in our study, the link is significant in attention problems, which does not interact with social adversity. In girls, the link is significant in aggression and does exhibit significant interaction with social adversity. The fact that social adversity has different moderating effects on behavior could be a cultural consequence. It is well known that male-preference is still very much prevalent in China today, and consequently parents tend to give boys more attention and supportive care-giving than to girls. Additionally, girls in general are more sensitive to their social environment than boys. This suggests the possibility that girls may experience a wider variety of social adversity and that these effects may be more prominent in leading to observable outcomes, such as behavioral problems. Indeed, the girls in our sample did exhibit higher average social adversity than boys.

Lastly, we observed the Hb-externalizing behavior link to be particularly strong for girls with low social adversity. This result is very interesting because it suggests that for girls who experience high levels of social adversity, the behavioral problems they exhibit are mostly the product of early factors such as parental education and occupation and living conditions. However, for girls who experience low levels of social adversity, their behavioral problems are more directly the result of environmental and nutritional factors which give rise to biomarkers such as low hemoglobin levels. This result points to the possibility that the positive effects of having normal Hb levels on reducing externalizing behavior is thwarted by the negative environmental factors present when social adversity is high.

Lozoff et al. have put forth the theory that the negative behavioral outcomes observed in children who were anemic as infants are the result of the compounding negative effects of anemia over time through lack of social referencing and functional isolation [24]. Social referencing is an important process of emotional communication between the parent and the child by which children use information and cues they receive from their caregivers to regulate their emotions and guide subsequent behaviors [24,51,52,53,54]. It has been previously discussed that a lack of social referencing can lead to functional isolation, where children who lack social referencing early on are less likely to seek or receive supportive care-giving from their parents [42]. As a result, children who are functionally isolated exhibit reduced responsiveness, lower vocalization, and less exploratory behavior [55]. This theory points to the possible mechanism by which infant anemia leads to adverse behavior outcome in early childhood, which leads functional isolation, which in turn further exacerbates behavioral problems in later childhood.

To our knowledge, these are the first findings to show that lower Hb levels in early childhood is associated with increased externalizing behavioral problems in Chinese preschool children. Our findings are amongst the first ones to report sex differences in the adverse effects of anemia on children’s externalizing behavior. Because of the inverse association between hemoglobin and later behavioral problems, nurses should regularly monitor children’s hemoglobin levels. Nurses should also educate parents on the benefits of a high-iron diet and encourage them to regularly incorporate high-iron foods, such as red meat and egg yolks, in children’s diets.

### Limitations

There are several limitations of this study which must be addressed. Firstly, our sample size of available Hb status is relatively small. Furthermore, the average Hb-levels (12.65 g/dL) of the children in our study would be considered fairly normal by most standards. For the purposes of analysis in this paper, we have used the 25th and 75th quartiles within our own data as grouping variables for Hb levels, so the children in the lowest Hb group in the present study may not be considered clinically anemic. Nevertheless, even within children with relatively normal levels of Hb, the link between Hb levels and externalizing behaviors can be observed. In addition, when stratified by sex, the sample sizes of the three subgroups of different Hb levels became smaller, notably the small number of the girls with low Hb levels (*n* = 7). Considering the possible insufficient power, we regarded a *p* value higher than 0.5 but less than 0.1 as trending significant. Despite the small sample size, we were able to find trending significant main effect of Hb and significant interactive effect of Hb levels and social adversity among girls, indicating the effect size is large enough to be detected with a small-size sample. However, we recommend future studies using larger sample size to replicate the study findings.

Secondly, it is unclear exactly what caused lower Hb levels for those children who exhibited it in our study. It is worth noting that the majority of the studies to date on anemia and children’s behavior uses Hb levels taken during the infantile or early toddlerhood years, while our present study took Hb measurements when then children were 4 years old. Therefore, it is unclear whether the children’s lower Hb levels at age 4 were carried over from infant anemia. Notwithstanding this ambiguity regarding the precise time of onset of anemia, the relationship between the children’s observed Hb levels at age 4 and externalizing behaviors at age 6 still holds.

Lastly, while not directly investigated in this paper, it is possible that lower Hb levels are a manifestation of overall malnutrition. Many papers in the past have established the link between early malnutrition (to which low Hb levels is a contributing component) and later behavioral problems in children, adolescents, and even adults [25,27,47,56]. However, our present study does not report other measurements for a more encompassing assessment of overall malnutrition. In addition, we did not include iron status in our analyses or other indicators such as child height and weight. A previous study did not find a significant association between iron status and childhood behavioral problems, but additional nutritional indicators—such as other micronutrients and biometrics—will be useful in separating the effects of Hb levels versus overall nutritional level [33]. Instead, the present study looked specifically at Hb levels in the hopes to begin the process to better identify which specific aspect of early malnutrition contributes to later behavioral problems. Future studies should include iron and other micronutrient statuses and/or deficiencies, along with biometrics, in the analysis in order to provide a multi-dimensional metric of childhood nutrition. In addition, future studies should also include internalizing behavior in addition to externalizing behaviors in order to better understand the broad spectrum of behavioral problems.

## 5. Conclusions

Our study on this sample of Chinese preschool children found that, firstly, lower Hb levels at age 4 is associated with higher scores in externalizing behavioral problems at age 6. Secondly, this relationship between Hb and externalizing behavior is not confounded by social adversity. Lastly, we observed sex differences in the link between Hb and behavioral problems in relation to social adversity. To our knowledge, these are the first findings to show that lower Hb in early childhood is associated with increased externalizing behavioral problems in Chinese preschool children. Our findings are amongst the first ones to report sex differences in the adverse effects of anemia on children’s externalizing behavior.

## Figures and Tables

**Table 1:** Descriptive statistics of all independent and dependent variables **^a^** included in analyses.

Variable	Total Group (*N* = 98)			Boys (*N* = 57)			Girls (*N* = 41)		
	Mean	SD	Range	Mean	SD	Range	Mean	SD	Range
Blood Hb-level **^b^** (g/dL)	12.65	0.91	10.00–15.40	12.55	0.95	10–15.40	12.78	0.83	11–14.20
CBCL Attention Score **^c^**	3.07	1.71	0–7	3.42	1.70	0–7	2.74	1.79	0–6
CBCL Aggression Score **^c^**	9.90	5.73	0–30	10.93	5.93	0–30	9.07	5.77	0–21
CBCL Externalizing Behaviors Score **^c^**	12.97	6.90	0–37	14.35	7.08	0–37	11.81	7.04	0–26
Social adversity **^d^**	3.04	2.06	0–11	2.96	2.18	0–11	3.21	1.90	0–8

**^a^** Includes only the 98 subjects with complete data on all independent (blood Hb levels, social adversity) and dependent variables (CBCL scores); **^b^** Measured at Age 4; **^c^** Measured at Age 6; higher score indicates more behavioral problems; **^d^** Measured at Age 6; higher score indicates higher social adversity.

**Table 2:** Demographic measures among children stratified by hemoglobin levels ^**a**^ at age 4.

Demographic Variable	<25th Percentile (*N* = 27)			25–75th Percentile (*N* = 48)			>75th Percentile (*N* = 25)			Analysis			
	% of Sex Group ^c^	Mean	SD	% of Sex Group ^c^	Mean	SD	% of Sex Group ^c^	Mean	SD	*X*^2^	*F*	df	*p*
Sex										5.723		2	0.057
Male	20.2			26.0			10.6						
Female	7.69			20.2			15.4						
Social adversity **^b^**		2.70	1.61		3.04	2.04		3.40	2.48		0.741	95, 2	0.479

**^a^** Hemoglobin levels are defined with cut offs of the 25th and 75th percentiles; **^b^** Taken at age 4; Range = 0–11; 11 = greatest severity; **^c^** % of sex group is defined to be, for each sex, the number of children with a specific hemoglobin level as a percent of the entire sample (*N* = 98) e.g., those in the less than 25th percentile hemoglobin level account for 20.2% of all males and 7.69% of all females in the sample.

**Table 3:** Univariate and multivariate analysis of variance **^a^** of the effect of hemoglobin levels **^b^** at age 4 on externalizing behavior at age 6.

*N* = 98	Main Effect: Hemoglobin			Effect Size	Moderator Effects:							
					Sex Interaction				Social Adversity ^c^ Interaction			
	*F*	df	*p*		*F*	df	*p*	Effect Size	*F*	df	*p*	Effect Size
Multivariate	2.016	4, 190	0.094 *	0.041	2.298	4, 184	0.061 *	0.048	1.496	4, 184	0.205	0.031
Univariate												
Attention	2.651	2, 95	0.076 *	0.053	3.346	2, 92	0.040 **	0.068	1.594	2, 92	0.209	0.033
Aggression	3.617	2, 95	0.031 **	0.071	1.221	2, 92	0.300	0.026	1.393	2, 92	0.253	0.029

**^a^** ANOVA and MANOVA were conducted using the attention problems and aggression subscales as the dependent variables; **^b^** Hemoglobin levels are defined with cut offs of the 25th and 75th percentiles; **^c^** Measured at age 4; high social adversity is defined as above 50th percentile in on 12-point social adversity scale. * Trending significant: 0.05 < *p* ≤ 0.1; ** Significant: *p* ≤ 0.05.

**Table 4:** Sex-stratified univariate and multivariate analysis of variance **^a^** of the effect of hemoglobin levels **^b^** at age 4 on externalizing behavior at age 6.

Sex, Type of Analysis, and Behavior Variable	Main Effect: Hemoglobin				Effect Size	Moderator Effects:				
						Social Adversity ^c^ Interaction				
	Mean (SD)	*F*	df	*p*		Mean (SD)	*F*	df	*p*	Effect Size
Boys (*N* = 57)	12.55 (0.95)					2.96 (2.18)				
Multivariate		1.780	4, 108	0.138	0.062		0.981	4, 102	0.422	0.037
Univariate										
Attention		3.387	2, 54	0.041 **	0.111		0.340	2, 51	0.713	0.013
Aggression		0.745	2, 54	0.480	0.027		1.209	2, 51	0.307	0.045
Girls (*N* = 41)	12.78 (0.83)					3.21 (1.90)				
Multivariate		2.200	4, 76	0.077 *	0.104		2.061	4, 40	0.095 *	0.105
Univariate										
Attention		1.617	2, 38	0.212	0.078		3.283	2, 35	0.049 **	0.158
Aggression		3.601	2, 38	0.037 **	0.159		2.797	2, 35	0.075 *	0.138

**^a^** ANOVA and MANOVA were conducted using the attention problems and aggression subscales as the dependent variables; **^b^** Hemoglobin levels are defined with cut offs of the 25th and 75th percentiles; **^c^** Measured at age 4. * Trending significant: 0.05 < *p* ≤ 0.1; ** Significant: *p* ≤ 0.05.

**Table 5:** Social adversity-stratified **^a^** results of univariate and multivariate analysis of variance **^b^** of the effect of hemoglobin levels **^c^** at age 4 on girls’ externalizing behavior at age 6.

Social Adversity, Type of Analysis, and Behavior Variable	Mean (SD)	Main Effect: Hemoglobin			Effect Size
		*F*	df	*p*	
Low Social Adversity (*N* = 24)	12.65 (0.81)				
Multivariate		3.936	4, 42	0.008 **	0.273
Univariate					
Attention		5.495	2, 21	0.012 **	0.344
Aggression		5.404	2, 21	0.013 **	0.340
High Social Adversity (*N* = 17)	13.04 (0.81)				
Multivariate		0.357	4, 28	0.837	0.049
Univariate					
Attention		0.448	2, 14	0.647	0.060
Aggression		0.474	2, 14	0.632	0.063

**^a^** Measured at age 4; high social adversity is defined as above 50th percentile in a 12-point social adversity scale; **^b^** ANOVA and MANOVA were conducted using the attention problems and aggression subscales as the dependent variables; **^c^** Hemoglobin levels are defined with cut offs of the 25th and 75th percentiles; ** Significant at two-tailed α = 0.05.
